# Semi-automated motor hotspot search (SAMHS): a framework toward an optimised approach for motor hotspot identification

**DOI:** 10.3389/fnhum.2023.1228859

**Published:** 2023-12-18

**Authors:** Desmond Agboada, Mirja Osnabruegge, Roman Rethwilm, Carolina Kanig, Florian Schwitzgebel, Wolfgang Mack, Martin Schecklmann, Wolfgang Seiberl, Stefan Schoisswohl

**Affiliations:** ^1^Institute of Psychology, University of the Bundeswehr Munich, Neubiberg, Germany; ^2^Department of Psychiatry and Psychotherapy, University of Regensburg, Regensburg, Germany; ^3^Institute of Sport Science, University of the Bundeswehr Munich, Neubiberg, Germany; ^4^Department of Electrical Engineering, University of the Bundeswehr Munich, Neubiberg, Germany

**Keywords:** motor hotspot, cortical excitability, neuronavigated TMS, motor mapping, grid system, TMS-cobot, SAMHS

## Abstract

**Background:**

Motor hotspot identification represents the first step in the determination of the motor threshold and is the basis for the specification of stimulation intensity used for various Transcranial Magnetic Stimulation (TMS) applications. The level of experimenters’ experience and the methodology of motor hotspot identification differ between laboratories. The need for an optimized and time-efficient technique for motor hotspot identification is therefore substantial.

**Objective:**

With the current work, we present a framework for an optimized and time-efficient semi-automated motor hotspot search (SAMHS) technique utilizing a neuronavigated robot-assisted TMS system (TMS-cobot). Furthermore, we aim to test its practicality and accuracy by a comparison with a manual motor hotspot identification method.

**Method:**

A total of 32 participants took part in this dual-center study. At both study centers, participants underwent manual hotspot search (MHS) with an experienced TMS researcher, and the novel SAMHS procedure with a TMS-cobot (hereafter, called cobot hotspot search, CHS) in a randomized order. Resting motor threshold (RMT), and stimulus intensity to produce 1 mV (SI1mV) peak-to-peak of motor-evoked potential (MEP), as well as MEPs with 120% RMT and SI1mV were recorded as outcome measures for comparison.

**Results:**

Compared to the MHS method, the CHS produced lower RMT, lower SI1mV and a trend-wise higher peak-to-peak MEP amplitude in stimulations with SI1mV. The duration of the CHS procedure was longer than that of the MHS (15.60 vs. 2.43 min on average). However, accuracy of the hotspot was higher for the CHS compared to the MHS.

**Conclusions:**

The SAMHS procedure introduces an optimized motor hotspot determination system that is easy to use, and strikes a fairly good balance between accuracy and speed. This new procedure can thus be deplored by experienced as well as beginner-level TMS researchers.

## 1 Introduction

Transcranial magnetic stimulation (TMS) is a non-invasive brain stimulation technique utilizing changing magnetic fields to electrically excite neuronal membranes and modulate changes in cortical networks through electromagnetic induction (Rossi et al., 2021; Siebner et al., 2022). Since its introduction in 1985 by Barker and colleagues (Barker et al., 1985), there has been many advancements in its engineering (Peterchev et al., 2014), and applications (Rossini et al., 2015; Nasr et al., 2022; Jannati et al., 2023), including but not limited to accurate targeting of stimulation locations via many different methods (Vucic et al., 2023).

Many different TMS stimulation protocols were developed in the motor cortex (Rossini et al., 2015). Here, a reliable output measure of stimulation is the measurement of corticospinal excitability from proximal muscles recorded with Electromyography (EMG) (Rossini et al., 2015; Siebner et al., 2022). For accurate localizations of the motor cortical representations of the muscle of interest, the TMS coil is carefully navigated over a broad area on the scalp representing the motor cortex, either manually by an experimenter holding the handle of the coil or with the aid of a stationary coil holder or even via a dynamic robotized arm (Ruohonen and Karhu, 2010; Romero et al., 2011; Rossini et al., 2015; Lefaucheur, 2019). The location in the motor cortex where a given stimulation intensity produces maximal peak-to-peak motor-evoked potentials (MEP) is defined as the motor hotspot (Rothwell et al., 1999; Rossini et al., 2015). This location is then marked, either physically on the participants’ scalp or digitally via a neuronavigation software. The process of finding a motor hotspot is, however, not that trivial. Depending on experience, most experimenters can find the motor hotspot within a few minutes or less, while a novice experimenter might take considerably longer times (van de Ruit et al., 2015). Moreover, the motor hotspot determination in a longitudinal or multisession study in the absence of a neuronavigation software can be quite challenging, as the motor hotspot can change between sessions sometimes by several millimeters (Julkunen et al., 2009; Caulfield et al., 2022).

There exist many different approaches for motor hotspot determination (for review, see Sondergaard et al., 2021). These include manual (pseudo-random navigation) (Pitkänen et al., 2015; van de Ruit et al., 2015), the use of landmarks (Malcolm et al., 2006), grid systems with or without magnetic resonance imagining (MRI)-guided neuronavigation (Brasil-Neto et al., 1992; Weiss et al., 2013), gridless-MRI- or functional magnetic resonance imagining (fMRI)-navigated TMS (Kallioniemi et al., 2016; Meincke et al., 2016, 2018), and more recently computational and closed-loop approaches that rely on electric field modeling for example, for mapping (Meincke et al., 2016; Tervo et al., 2020; Nieminen et al., 2022; Numssen et al., 2023; Weise et al., 2023). While using landmarks, and other manual navigation methods were fairly good for quick and reliable hotspot determination (Malcolm et al., 2006; Pitkänen et al., 2015; van de Ruit et al., 2015) a higher level of accuracy might usually be sacrificed for speed (Julkunen et al., 2009). However, the opposite also applies for the more robust closed-loop, as well as novel computational methods which sought to increase accuracy and precision of motor hotspot determination (Meincke et al., 2016; Harquel et al., 2017; Weise et al., 2023). While excellent mappings were achieved, it remains to be seen if these innovative methods would be useful for non-longitudinal single session experiments. As demonstrated in a recent study, more than 24 h were required to achieve precision and individualized motor mapping – 2 h for manual preparation, 10 h for automated head model construction, 2 h of TMS session, and 12 h of computational post-processing (Weise et al., 2023). This does not include the high level of expertise needed for the computational modeling and general knowledge of computer programming. This speed-precision trade-off presents a conundrum, making it somewhat difficult to solely recommend one paradigm over the other.

Grid systems of motor hotspot determination have been the earliest attempts at optimization (Wassermann et al., 1994; Classen et al., 1998), and currently the middle ground between pseudo-random navigational manual methods and the computationally advanced precision-based methods (Romero et al., 2011; Weiss et al., 2013). Compared to manual methods, grid procedures ensure that areas in the motor cortex are more systematically mapped. However, the lack of a standardized grid system has led to suboptimal grid sizes, and spacings which could be accountable for heterogeneous results in the literature (Sondergaard et al., 2021). High amounts of grid points for example increase the time to navigate the entire grid area which may lead to experiment-induced participant fatigue, a well-known extraneous variable in TMS research (Ridding and Ziemann, 2010). Furthermore, finding the center of gravity (CoG) of a grid requires navigating through each grid point, and computations of the motor hotspot via the calculations of the map area and volume are often done offline, which also prolongs experiment time (Sondergaard et al., 2021).

Recently, Giuffre et al. (2021) investigated the reliability of robotic TMS mapping of the first dorsal interosseous (FDI) muscle in the left primary motor cortex using a 12 by 12 grid system (7 mm spacing) paired with neuronavigation. They analyzed map characteristics such as the area, volume, CoG, and hotspot magnitude offline, and found a good to excellent short and long-term reliability for all three measures (24 h and 4 weeks) (Giuffre et al., 2021). Here too, the reliance on CoG, as well as the use of a larger grid area of 5,929 mm^2^ increased the experimental time and computation needed to access the motor hotspot within this large area. While CoG for determining motor hotspots are usually reliable, some recent studies have found but low absolute reliability of CoG for three common muscle cortical representations in the motor cortex (Nazarova et al., 2021).

With the present feasibility study, we propose a standardized and a more intuitive semi-automated motor hotspot search procedure (SAMHS) for finding the motor hotspot using MRI-guided robotized neuronavigation for a time-efficient motor mapping. Within this framework, the emphasis is placed heavily on striking a careful compromise between speed and accuracy of finding the motor hotspot. Thus, we believe this may be the most efficient automation of motor hotspot determination with a fairly minimal input from the experimenter. The knowledge required to use this procedure is relatively small compared to the training and the experience needed for other forms of motor hotspot determination, such as the classical landmark, pseudo-random, and the more computationally rigorous novel methods. Furthermore, in this feasibility study, we aim to test the practicality and accuracy of SAMHS by a comparison with a manual motor hotspot identification method, using a dual-center study design.

## 2 Materials and methods

The present experiment was conducted at the Center for Neuromodulation of the University of Regensburg, Germany (RGB) and at the Institute of Psychology of the University of the Bundeswehr Munich, Germany (MUC). The applied experimental procedures were approved by the respective local ethics committees. All participants gave written informed consent prior to study participation, in accordance with the Declaration of Helsinki.

### 2.1 Participants

Participants were recruited according to the following inclusion criteria at both sites: right-handedness (self-reported), aged 18–50 years; no contraindications to TMS (implants, epilepsy or traumatic brain injury); absence of severe neurological or psychiatric diseases; no addictions to psychoactive substances; and no central nervous system medications. At the study site in RGB, 12 healthy subjects (7 females) aged between 21 and 42 years (25.6 ± 5.8 years) were eligible and participated in the experiment. In the MUC study site, 20 subjects between 18 and 28 years (22.8 ± 2.9 years) met the inclusion criteria and participated in the study. Hence a total number of 32 subjects (14 females; 23.8 ± 4.5 years) participated in the present study. All subjects received monetary compensation for their participation.

### 2.2 Transcranial magnetic stimulation

At both study sites, a MagVenture X100 TMS device (MagVenture, Farum, Denmark) delivered single TMS pulses at about 0.25 Hz (with a 10% jitter) through a standard 60 mm figure of 8 coil (uncooled B60 for MHS, and cooled AP65 coils for CHS). For both manual, and TMS-cobot stimulation conditions, the coil was positioned tangentially over the motor cortical region at an angle of 45 degrees to the sagittal midline, with the handle pointing backward, and current flowing in the anterior-posterior-posterior-anterior direction (AP-PA) in the coil. The maximum magnetic flux density delivered by the device through each of the coils was 1.4 Tesla. All stimulations were delivered in biphasic pulse shape in the standard mode configuration (pulse width = 280 μs).

### 2.3 Neuronavigated TMS collaborative robotized coil holder (TMS-cobot)

Both centers of study used the TMS-collaborative robotized coil holder, TMS-cobot (Axilum Robotics, Schiltigheim, France), which has integration and support for the MagVenture cooled AP65 coil, and the Localite (Localite GmbH, Bonn, Germany) neuronavigation software. The TMS-cobot has built-in safety features utilizing collision detection mechanisms at each robotic joint, which allows for safe navigation during fully automated and manual modes. The pressure sensors embedded in the encapsulated AP65 coil ensured good contact with head and coil. Using optical trackers placed on the participants’ forehead, the cobot was able to maintain the position and orientation of the coil in the hemispherical space during each experimental session. Slow head movements of the participant were well compensated for due to real-time tracking, while sharp or sudden head movements disengaged the coil for safety reasons. The cobot has 6 degrees of freedom in robotic arm adjustment. In terms of accuracy of positioning, the cobot has below 2 mm in arm accuracy and repeatability, within a half-hemispheral working space (Axilum Robotics, Schiltigheim, France).

Neuronavigation of TMS coils (B60, and AP65) in the motor cortex was carried out by the Localite Neuronavigation system, Robotic Edition (Localite GmbH, Bonn, Germany) using the Polaris camera (Polaris Spectra, NDI, Waterloo, Canada) at both research centers. It is important to note that though the Robotic Edition of the Localite neuronavigation system was used for navigating both coils, only the AP65 cooled coil has TMS-cobot integration.

For both study centers, individual T1-weighted structural MRI images acquired from a 3 Tesla MRI Siemens scanner (Siemens, Munich, Germany) at the Department of Psychiatry and Psychotherapy, Ludwig Maximilian University Munich and the Department of Psychiatry and Psychotherapy, University of Regensburg, Germany were used. T1-weighted MUC: MPRAGE with 176 sagittal slices, matrix size = 256 × 240, voxel size = 1 mm^3^, flip angle 9°, TR/TE/TI = 2300/4.16/900 ms. T1-weighted RGB: MPRAGE 160 slices, 256 × 256, voxel size = 0.977 × 0.977 × 1 mm^3^, flip angle 9°, TR/TE/TI = 1910/3.67/1040 ms.

### 2.4 EMG recording and preprocessing

Electromyogram data were recorded using the Brain Vision Recorder Version 1.24.0101 software with a BrainAmp DC system in combination with a BIP2AUX adapter (all Brain Products GmbH, Germany) (RGB) and a Digitimer D360-R amplifier (Digitimer, Welwyn Garden City, Hertfordshire, UK) with a CED 1401 A/D converter (Cambridge Electronic Design, Cambridge, UK) (MUC). The muscle activity of the FDI muscle of the right hand was recorded using a bipolar belly-tendon montage with the ground placed over processus styloideus ulnaris. The same gel electrodes with Ø 24 mm (Kendall™ H124SG, Cardinal Health, USA) were used at both centers. Prior to recording, the skin was primed with a skin preparation gel (Weaver and Company, Aurora, Colorado, USA) and cleaned with alcohol. The sampling rate was 10 kHz (down-sampled to 5 kHz) (RGB) and 5 kHz (MUC).

In RGB, the pre-processing was done with custom written scripts in MATLAB (version 2020b; Natick, Massachusetts, USA) using the EEGLAB toolbox (Delorme and Makeig, 2004) with the TMS-EEG Signal Analyzer extension (Rogasch et al., 2017) and the Fieldtrip toolbox (Oostenveld et al., 2011). In MUC, the MEPs were recorded and processed using CED Signal Software version 7.07 (Cambridge Electronic Design Limited, Cambridge, UK), and analyzed using custom written MATLAB scripts (MATLAB version 2021b; Mathworks, Natick, Massachusetts, USA). Both centers applied a 4th order Butterworth band-pass filter between 10–500 Hz.

Individual trials were visually screened and discarded if they did not contain MEP with peak-to-peak amplitude of at least 50 μV or contained artifacts such as muscle pre-activations of about 25 μV from baseline 50 ms before the pulse or 100 μV 300–100 ms before the pulse (Bigoni et al., 2022). A total of 2.9% (RGB) and 0.6% (MUC) of the recorded MEPs were rejected for artifact contamination. Further, the MEP-to-baseline ratio (MEP_*p*2*p*_/baseline_*p*2*p*_) was calculated and trials with less than 160% of the ratio were automatically excluded at the RGB study center. The peak-to-peak amplitude was calculated in a time interval of 10–40 ms after the pulse. The latency of the MEP onset was defined as the time point where the absolute differential of the EMG signal exceeded the absolute mean differential during the MEP window (10–40 ms post pulse, MUC) and the time point where the first post-stimulus EMG peak signal exceeds ± 2 SD of the mean baseline activity 200–100 ms before the pulse (RGB).

### 2.5 Experimental procedure

**FIGURE 1:**
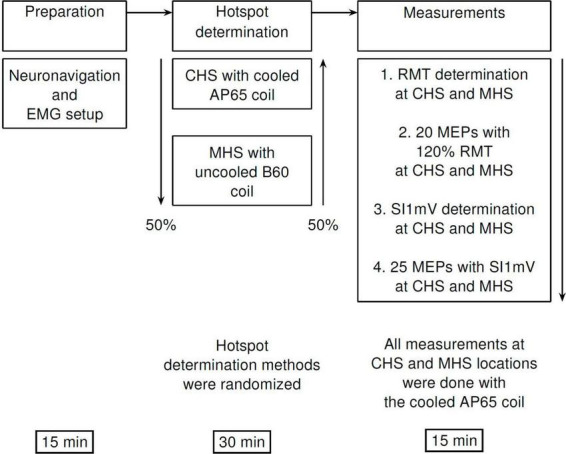
Diagrammatic representation of the experimental procedure. First, participants were comfortably seated in an experimental chair, and neuronavigation setup as well as EMG electrode placements were done. This was followed by motor hotspot determination—via the manual (MHS) or the cobot (CHS) methods using the B60 and the AP65 coils, respectively. Finally, measurements of RMT, SI1mV were made, and MEP measurements with 120% RMT (20 MEPs), and SI1mV (25 MEPs) were recorded at CHS, and MHS locations. All recordings of MEPs at both hotspot locations were made with the AP 65 coil to avoid any differences in measurement due to coil characteristics. MHS, manual hotspot search; CHS, cobot hotspot search.

Motor hotspot search methods (MHS and CHS) were randomized *a priori*. All measurements from the MHS and CHS locations were performed in the following order – RMT, MEPs induced by 120% of RMT (MEP_120%*RMT*_), SI1mV, and MEPs produced using SI1mV (MEP_*SI*1*mV*_) (Figure 1). After arrival in the lab, participants were seated comfortably in an adjustable treatment chair and had EMG electrodes fixed on their hand (see Section “2.4 EMG recording and preprocessing”) as well as an optical tracker to the right forehead for robot-assisted neuronavigation [see Section 2.3 Neuronavigated TMS collaborative robotized coil holder (TMS-cobot)”]. Throughout the entire experiment participants were instructed not to move as well as to relax their right hand.

#### 2.5.1 The MHS method

For the MHS procedure, at the RGB study center, we targeted the C3 electrode position (10-20 EEG system), while at the MUC study center, we targeted both the C3 electrode position (10-10 EEG system) as well as the location for the FDI muscle, and used the online MEPs as a guide during the navigation of the TMS coil over the scalp. At both study centers, the handle of the B60 coil was held by the experimenter and oriented backward at approximatively 45° to the sagittal midline. With an initial intensity of 45% maximum stimulator output (MSO) single pulses were applied at different locations around C3 until a point was identified where MEPs of the FDI muscle were stable, and in the case of MUC center, FDI locations were targeted as well. With the MHS method, stimulus intensities, coil positions as well as angulations were manually adjusted by the experimenter to optimally discriminate between hotspots. To control for inter-rater variability, only one experimenter at each study site carried out all manual hotspot determinations. At both centers, the experimenter conducting the MHS method had more than a year of experience in TMS research (RGB = 1.5 years, MUC = 7 years).

#### 2.5.2 The SAMHS (CHS) method

The grid sizes and spacings were selected after careful review of literature and rigorous piloting with a few participants. The robotized neuronavigated TMS (TMS-cobot) ensured a very high accuracy of coil movements, eliminating investigator errors due to coil handling (de Goede et al., 2018).

**FIGURE 2:**
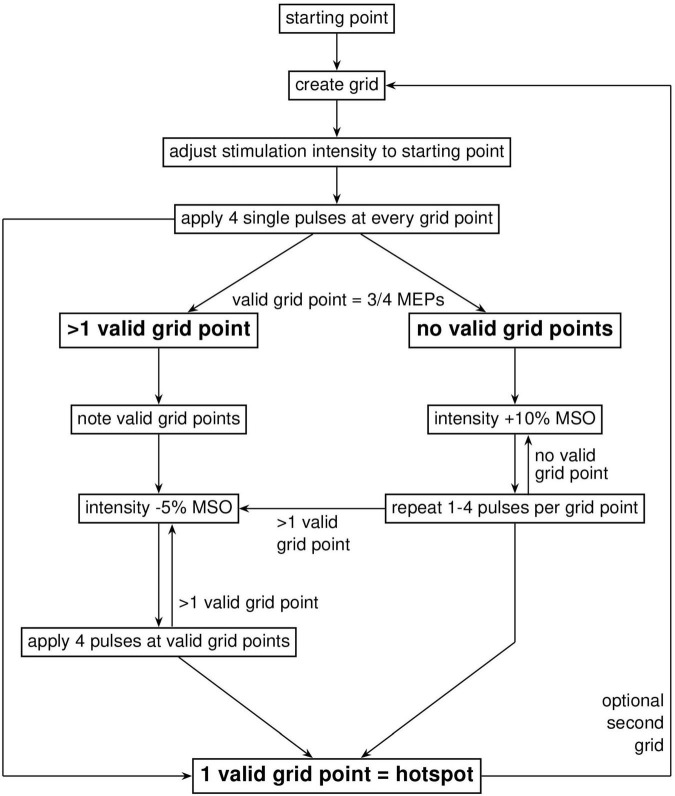
Workflow diagram of the cobot hotspot search (CHS) procedure. Diagram shows the step-by-step procedure to identify the motor hotspot with the CHS procedure. First, a coarse 4 × 4 two-dimensional square grid with 10 mm spacing was placed around the FDI MNI coordinates (*x* = –34.19, *y* = –14.33, *z* = 66.83), after which navigation started according to a systematic procedure shown in the figure. When the hotspot is found in the big grid, no further action was needed. To increase resolution, and therefore accuracy or validation of the motor hotspot, a second optional 3 × 3 grid with 5 mm spacing is recommended. Here, navigation through the finer 3 × 3 grid to localize the motor hotspot is similar to the big 4 × 4 grid.

The first step of the CHS procedure was to define a coarse 4 × 4 grid with 10 mm spacing around the mean FDI hotspot in the MNI space, using the FDI MNI coordinates found by Numssen and colleagues, *x* = −34.19, *y* = −14.33, *z* = 66.83 (Numssen et al., 2021). The grid was oriented at 0° to the longitudinal fissure (midline). The mean FDI hotspot was then used as the starting point with a coil rotation of 45° to the midline and an intensity of 45% MSO. Single pulses with an inter-stimulus interval of 4 s (10% jitter) were applied at this starting position. If no MEPs were detected in the recording software, the intensity was increased in steps of 10% MSO up to a maximum of 3 times (75% MSO). Either way, if MEPs were present or 75% MSO was reached, the experimenter started navigating through the 4 × 4 grid, delivering 4 pulses at each grid point. We then noted the grid points which were most stable (at least 3 out of 4 MEPs) and had the highest amplitude. In case multiple points were identified, intensity was decreased by 5% MSO and the individual points were compared against each other until the most stable one was found. Figure 2 provides a step-by-step overview of the CHS procedure. Further details can be found in the Supplementary materials.

To improve the spatial accuracy of this potential hotspot location, we applied a finer 3 × 3 grid, with 5 mm spacing and an orientation of 0° to the midline. The finer grid was placed around the best point of the initial 4 × 4 grid. We went through the same procedure as before, with 4 pulses at each point until the best point is identified in this fine grid. This location is designated as the motor hotspot.

#### 2.5.3 Resting motor threshold determination and measurements

After motor hotspot determination, the resting motor threshold (RMT), that is the lowest stimulation intensity needed to elicit MEPs of at least 50 μV in at least 50% of trials for both hotspots was measured (Rossini et al., 1994, 2015; Rothwell, 1997). Ten (10) single pulses of TMS were applied with an ISI of 4 s (10% jitter), and RMT was identified as the stimulus intensity that produces 5 out of 10 MEPs of at least 50 μV peak-to-peak. Subsequently we applied 20 single pulses with an inter-stimulus interval of 4 s with an intensity of 120% of the RMT (MEP_120%*RMT*_). Afterward we identified the intensity that elicits MEP peak-to-peak amplitudes of about 1 mV (range: 0.85–1.15 mV) (SI1mV) and recorded 25 MEPs with this intensity (MEP_*SI*1*mV*_). For RMT, SI1mV as well as subsequent MEP recordings, the same cooled AP65 coil used.

### 2.6 Statistical analysis

Data from both study sites were pooled for statistical analysis. Analyses were performed with the statistics software R (R version 4.0.3; R Foundation for Statistical Computing, Vienna, Austria) using the packages “lme4” and “ggplot2.” Differences between hotspot determination methods (CHS vs. MHS) were analyzed with linear-mixed effect models for each outcome parameter of interest: RMT (% MSO), SI1mV (% MSO), mean and standard deviation of MEP peak-to-peak-amplitude (μV) and MEP latency (ms) separately for RMT and SI1mV. The hotspot determination method was always treated as a fixed effect (CHS/MHS) and subject was always treated as a random effect in each model. Models were adjusted for the following covariates: sex (male/female), age (years) and study center/site (RGB/MUC) – *lmer(outcome* ∼ *method* + *sex* + *age* + *site* + *(1 | subject)*). Statistical significance was set at the 5% level for all analysis. Additionally, the duration needed for the determination of the motor hotspot for both methods was reported descriptively.

### 2.7 Target projection

**FIGURE 3:**
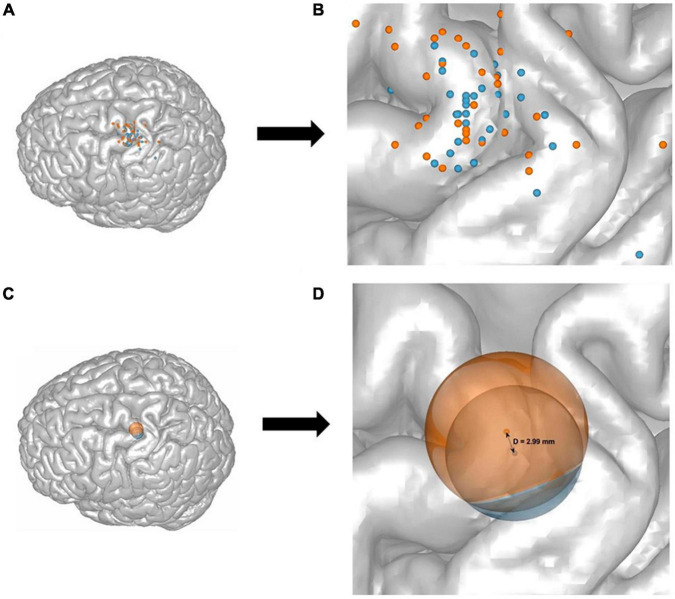
A representation of all 32 participants’ target points of the CHS (blue) and the MHS (orange) hotspots, and their mean and standard deviation projected onto the stereotypical N27 Collins brain **(A,C)**, as well as a zoom-in illustration **(B,D)**. Each dot represents one participants’ motor hotspot according to the CHS (blue) and MHS (orange) procedures **(A,B)**. The mean and standard deviation of all participants target points were calculated and shown as two spheres. The groupwise means are the centers of the spheres and the dispersion (or SD) is represented as the radius **(C,D)**. The projected distance between the means of CHS, and MHS was *D* = 2.99 mm (SD_CHS_ = 8.86 mm, and SD_MHS_ = 10.49) **(D)**.

For the purpose of comparable visualization, the individual and mean CHS and MHS hotspot locations were projected to the surface of one single brain template. To create the projection, the common “Colin27_T1_seg_MNI” was used as a template. The image was loaded in Matlab (version R2021a) and with a custom written script, and a geometric central point was calculated and used as an origin. A line was generated between the origin and each target point for the individual hotspots in MNI space. The surface projected points are the points where the lines and the surface of the brain overlap. Each projected point was visualized by a sphere with a radius sufficient for a visible distinction of single points, with the midpoint at the projected target point (Figures 3A, B). For the group-average projection of MHS and CHS, the same method was used. Here, however, the centers of the spheres represent the mean while the radii represent the standard deviation for both CHS and MHS methods.

## 3 Results

All participants tolerated the stimulation well, and there were no reports of any discomfort or adverse side-effects.

**FIGURE 4:**
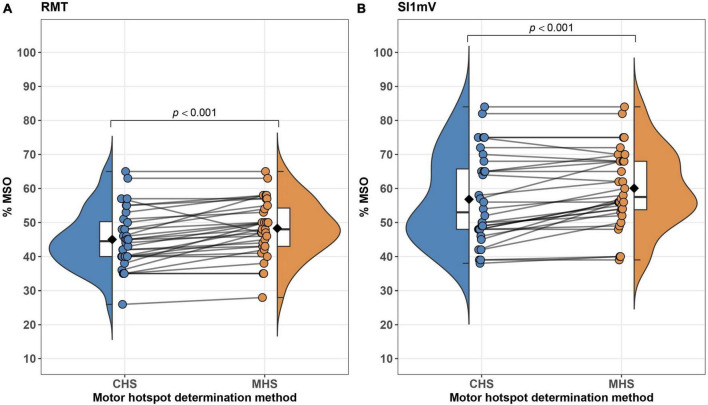
Differences between MHS and CHS methods on an average–(box plots embedded in violin plots) as well as at the subject-level (points connected with lines) are illustrated. Significant differences between MHS and CHS were observed with the CHS exhibiting lower% of MSO for RMT **(A)** and SI1mV **(B)**. RMT, resting motor threshold; MSO, maximum stimulator output; SI1mV–stimulus intensity required for 1 mV peak-to-peak MEP amplitude.

Linear mixed effect models adjusted for sex, age and study site revealed significant differences between MHS and CHS for the outcome parameters RMT and SI1mV, with the CHS method producing a lower percent of MSO for both measures [RMT: *t*_(32.00)_ = 4.49, *p* < 0.001; SI1mV: *t*_(32.00)_ = 4.20, *p* < 0.001]. Average differences together with individual subject differences between MHS and CHS for RMT and SI1mV (Figure 4). Further, a statistical trend toward a higher MEP_*SI*1*mV*_ amplitude for the CHS approach was observed. No statistically significant differences between MHS and CHS were observed for MEP_120%*RMT*_ amplitudes as well as MEP_120%*RMT*_ and MEP_*SI*1*mV*_ latencies. Likewise, no differences between the two hotspot search methods in the variability (SD) of MEP_120%*RMT*_ and MEP_*SI*1*mV*_ amplitude and latency were present. Descriptive as well as inferential statistical differences between MHS and CHS are shown in Table 1. The average motor hotspot determination time was 15.60 min for the CHS (SD = 2.52; Min = 10.95; Max = 21.80) and 2.43 min for the MHS (SD = 1.33; Min = 0.42; Max = 4.82). In 9 out of the 32 participants, the subject’s motor hotspot was obtained in the bigger 4 × 4 grid.

**TABLE 1:** Descriptive and inferential statistics of MHS and CHS methods.

	M ± SD	Estimate	T _(df_, _se)_	*p*
**RMT (% MSO)**				
MHS–CHS	48.28 ± 8.37 – 45.00 ± 8.79	3.28	4.49 _(32.00,__ 0.73)_	**<0.001**
**SI1mV (% MSO)**				
MHS–CHS	60.06 ± 11.41 – 56.81 ± 13.19	3.25	4.20 _(32.00,__ 0.77)_	**<0.001**
**MEP_120%*RMT*_ amplitude (μV)**				
MHS–CHS	1083.72 ± 1101.36 – 1008.69 ± 692.14	75.02	0.75 _(32.00,__100.40)_	0.460
**MEP_SI1mV_ amplitude (μV)**				
MHS–CHS	941.79 ± 259.78 – 1036.32 ± 209.32	-94.53	−1.98 _(32.00,__47.41)_	0.056
**MEP_120%*RMT*_ latency (ms)**				
MHS–CHS	24.05 ± 1.88 – 24.02 ± 1.77	0.03	0.27 _(32.00,__ 0.14)_	0.786
**SI1mV–MEP latency (ms)**				
MHS–CHS	24.30 ± 1.64 – 24.12 ± 1.52	0.18	1.33 _(32.00,__ 0.14)_	0.192
**MEP_120%*RMT*_ amplitude (μV)–SD**				
MHS–CHS	510.37 ± 355.60 – 518.24 ± 316.14	-7.87	−0.18 _(32.00,__43.96)_	0.859
**MEP_SI1mV_ amplitude (μV)–SD**				
MHS–CHS	509.22 ± 222.41 – 522.52 ± 227.84	-14.00	−0.55 _(32.00,__24.21)_	0.586
**MEP_120%*RMT*_ latency (ms)–SD**				
MHS–CHS	0.87 ± 0.66 – 0.79 ± 0.51	0.08	0.81 _(32.00,__ 0.10)_	0.423
**MEP_SI1mV_ latency (ms)–SD**				
MHS–CHS	0.79 ± 0.56 – 0.69 ± 0.48	0.09	0.91 _(32.00,__ 0.10)_	0.378

The table shows RMT, SI1mV, and MEPs recorded with SI1mV and 120% RMT of the MHS, and the CHS methods, and the linear mixed effects regression statistics computed on the comparisons of measurements of these two methods. CHS, cobot hotspot search; MHS, manual hotspot search; RMT, resting motor threshold; MSO, maximum stimulator output; SI1mV, stimulation intensity to evoke MEPs of 1 mV; MEP, motor evoked potential; M, mean; SD, standard deviation; df, degrees of freedom; se, standard error.

The groupwise CHS coordinate points have an SD = [4.88 5.84 4.53], which equals 8.86 mm in length, and MHS has an SD = [6.29 7.48 3.79] which is 10.49 mm in length. The unprojected calculated Euclidean distance between the two hotspot methods is 3.29 mm. The projected Euclidean distance between both hotspots of all participants is 2.99 mm (Figure 4).

## 4 Discussion

The objective of this feasibility study was to develop a new framework of motor hotspot determination with the potential to overcome shortcomings of previous approaches such as lack of accuracy in manual motor mapping approaches or time-consuming and sophisticated computational modeling concepts. With the aid of an MRI-guided robot-assisted neuronavigated TMS system, we introduced a standardized and time-efficient grid-based motor mapping framework. Further, we tested the efficiency of this framework by a direct comparison with the well-established manual motor hotspot search procedure. Our main finding of this feasibility study was that in contrast to MHS, our new CHS approach demonstrated a statistically significant lower RMT and SI1mV values. Additionally, the duration of the CHS was, as expected, considerably longer than that of the MHS. No significant differences were observed for MEP_SI1mV_ and MEP_120%RMT_ for the CHS and MHS. All participants tolerated the stimulations very well, and no reports of any discomfort or adverse effects were observed.

### 4.1 RMT and peak-to-peak MEP amplitudes for MHS and CHS

In the current study, we found the CHS procedure produced a significantly lower RMT compared to the MHS. It could be argued that the grid method most accurately localized the motor hotspot, as the mapping was up to 5 mm inter-grid point distance, which should increase accuracy of mapping, compared to the manual navigation method. Though higher resolution was found to increase accuracy of motor hotspot (Weiss et al., 2013; de Goede et al., 2018), there could be an upper limit or threshold of map accuracy such that decreasing grid spacing did not further improve map accuracy beyond 5 mm (de Goede et al., 2018). In 9 of the 32 participants (28%) the bigger 4 × 4 grid (10 mm spacing) was sufficient for finding the motor hotspot. This confirms previous studies using the CoG grid technique that found reliable motor hotspots with the coarse grid spacing of 10 mm (Ward et al., 2016). For the lower RMT of the CHS compared to the MHS, lower TMS intensity eliciting comparatively higher peak-to-peak MEP amplitude is the definition of the motor hotspot (Rossini et al., 2015), which again means the CHS procedure might have localized a better hotspot than the MHS. Previous studies comparing the CoG grid system to the manual motor hotspot determination method did find a better mapping with a higher reliability for the CoG (Meincke et al., 2016), which is intuitive, as mapping with grid methods are superior to manual navigation (Sondergaard et al., 2021). Though in the MHS condition, the motor hotspot was found using the uncooled B60 coil versus the cooled AP65 coil used for the CHS condition, all measurements at both hotspots were done by the AP65 coil, with the CHS hotspot having lower RMT, and a trend-wise higher peak-to-peak MEP amplitudes obtained with SI1mV compared to the MHS. It could thus be argued that doing measurements with one coil type at both hotspot locations rules out differences due to coil characteristics such as the magnetic and the induced electrical fields in the brain.

No significant differences were found in the MEP amplitudes and latencies induced by 120% of RMT, and SI1mV for both CHS and MHS, except for the aforementioned trendwise effects for MEP_SI1mV_. The similarity in MEPs recorded from both hotspots likely reflects the accuracy of the MHS, which was not far away from the CHS (averaged projected distance = 2.99 mm). In fact, the mean of all participants’ MHS and CHS locations mostly overlapped (Figures 3C, D), though the MHS locations showed a higher dispersion from the mean when compared to the CHS locations. Additionally, since the RMT, and SI1mV of the MHS were significantly higher than that of the CHS, it could also be argued that the similarity of responses in MEP absolute values was due to the higher intensity at the ‘less optimal’ MHS location. This becomes even clearer when looking at the average individual unprojected Euclidean distance between the MHS and the CHS for each participant, where the two hotspots differ by 9.75 mm on average. It is already known that as you move farther away from the motor hotspot, to produce a similar response, stimulation intensity has to increase. A higher stimulation intensity of the MHS at a nearby location of the CHS would activate the same neuronal assembly thereby producing similar corticocortical excitability (Rossini et al., 2015; Siebner et al., 2022).

### 4.2 Duration of hotspot determination

To the best of our knowledge, this is the first study comparing motor hotspots accessed by a manual, and a robot-assisted method with a novel grid search approach.

In a previous investigation, which compared manual hotspot search and motor mapping with neuronavigation-assisted procedures, the manual paradigm was conducted significantly faster (Julkunen et al., 2009). As expected, the CHS needed substantially more time in contrast to MHS (15.60 min vs. 2.43 min, on average), which raises the question if it is worthwhile to invest significantly more time for motor mapping using the CHS. The relatively longer time needed to determine the motor hotspot with the CHS compared to the MHS should not be interpreted as a disadvantage. In fact, in most instances this longer time will be more helpful for new TMS researchers who would otherwise need many months of experience to use the MHS methods effectively. As already mentioned, more technologically advanced methods of motor mapping require even longer durations of many hours (Weise et al., 2023) which makes motor hotspot determination as a part of an experimental session nearly impossible.

In the clinic, where nurses, medical students, and other clinical staff are tasked to do experimental work, training these workers to use the SAMHS procedure will be much more beneficial vis-à-vis the cost and time needed for these clinical studies. Moreover, the computational considerations, which serve as a limitation for advanced motor mapping paradigms do not exist with the SAMHS method.

It should be noted here that cobot or other forms of robotized-TMS are not required for the SAMHS procedure. What is needed is a neuronavigated-TMS, which allows for a real-time navigation to coordinates pre-selected as the starting point of the grid. This means most TMS laboratories that do not yet have robotized-TMS but have a neuronavigation system can still use the SAMHS procedure.

### 4.3 CHS vs. CoG grid paradigms

As previously stated, traditional CoG grids used a large grid size which covers the whole motor cortex, and sometimes more than half of the entire hemisphere of interest (Sondergaard et al., 2021). This obviously introduces the added problem of time as there is navigation of each grid point until coordinates of the best response are identified. In contrast, the novel grid method presented here has a definite starting point – a coordinate of the FDI muscle in the MNI space (Numssen et al., 2023). This cuts out the need to put a large grid over a large portion of the hemisphere, reducing considerably time for grid navigation, and computations of grid center of gravity. Similarly, grid spacing is optimally selected – first, a large spacing of 10 mm which has been widely used in the CoG grid literature (Sondergaard et al., 2021), and then a finer spacing of 5 mm. Increasing the resolution did improve tremendously the hotspot location similar to other studies using the classical CoG methods (Weiss et al., 2013; de Goede et al., 2018). In 72% of participants in this study, the CHS procedure found better hotspot locations in the smaller 3 by 3 square grid of 5 mm spacing compared to the bigger 4 by 4 with 10 mm spacing.

### 4.4 Limitations and recommendations

There were a few limitations of this study. First, we did not compare this new grid method with the classical CoG grid technique, which is the *de facto* standard in the TMS grid literature. This was partly due to our research question which sought to compare the manual versus this novel grid techniques. A comparison between the classic CoG and this novel grid systems would have yielded more information about which grid system is better, in terms of time, and efficiency. Further studies in the future should systematically explore how this novel grid method compares with the classical grid method.

All measurements were made with the muscle at rest. We did not investigate active motor thresholds, nor measurements with muscle contraction. Recently, it was shown that motor mapping with muscles contracted shows good reliability (Kahl et al., 2023). There should be studies in the future to optimize and extend SAMHS to muscles under voluntary contraction.

Thirdly, we did not test this grid method with a non-neuronavigated or non-robotized TMS. This might be particularly needed for laboratories which do not have robotized TMS. While the methodology might be different – experimenter coil handling versus robot-assisted, in principle, the procedures of navigating through the grid in a systematic manner can still be applied, though quite challenging without neuronavigation. Here too, perhaps this method can be further optimized for such laboratories with no robot-assisted TMS.

Finally, this new grid system was validated in healthy young adult participants. Generally, we do not expect a significantly large change between age groups for simple motor mapping with TMS using a grid. However, for clinical populations, perhaps an individualized approach might be better. This could be due to significantly different anatomical and neurophysiological changes observed in clinical pathologies that are absent in healthy populations. Such brain changes might influence how motor mapping is done.

## 5 Conclusion

In this feasibility study, we introduced a novel grid system for motor hotspot determination, and compared its efficacy with a manual hotspot search procedure. This grid method is more efficient compared to the classic manual method as it has a clear starting point and standardized procedures for navigating through the grid, with the hotspot determined online. RMT and SI1mV were lower, and peak-to-peak MEP amplitudes obtained with SI1mV were higher trend-wise when compared with values obtained with the manual search method. Though it took relatively longer compared to the manual method, this standardized procedure makes up for the time, by striking a fairly good balance between speed and accuracy. Finally, the level of experience, and computational processes needed for this grid search method are comparatively low, in relation to other non-manual motor hotspot search approaches.

## Data availability statement

The raw data supporting the conclusions of this article will be made available by the authors, without undue reservation.

## Ethics statement

This study was approved by the Ethics Committees of the University of the Bundeswehr Munich, and the University of Regensburg. The study was conducted in accordance with the local legislation and institutional requirements. Participants provided their written informed consent to participate in this study.

## Author contributions

DA: conceptualization, methodology, investigation, formal analysis, visualization, writing—original draft, supervision, and project administration. MO: investigation, methodology, formal analysis, visualization, and writing—original draft. RR: investigation, methodology, formal analysis, and writing—original draft. CK: investigation, methodology, visualization, and writing—original draft. FS: formal analysis, visualization, and writing—review and editing. WM: funding acquisition and writing—review and editing. MS and WS: writing—review and editing. SS: conceptualization, methodology, formal analysis, visualization, writing—original draft, supervision, and project administration. All authors contributed to the article and approved the submitted version.
