# Supplementary material for: Semi-automated motor hotspot search (SAMHS): a framework toward an optimised approach for motor hotspot identification
Source: Front Hum Neurosci. 2023 Dec 18;17:1228859. doi: 10.3389/fnhum.2023.1228859 (PMC10757939; doi:10.3389/fnhum.2023.1228859)
Supplement: Supplementary file 1 [file Data_Sheet_1.docx]

**Semi-automated motor hotspot search (SAMHS): A framework towards an optimised approach for motor hotspot identification.**

Desmond Agboada^1*^, Mirja Osnabruegge^1,2^, Roman Rethwilm^3^, Carolina Kanig^1,2^, Florian Schwitzgebel^4^, Wolfgang Mack^1^, Martin Schecklmann^2^, Wolfgang Seiberl^3^ & Stefan Schoisswohl^1,2*^

^1^ Institute of Psychology, University of the Bundeswehr Munich, Neubiberg, Germany.

^2^ Department of Psychiatry and Psychotherapy, University of Regensburg, Regensburg, Germany.

^3^ Institute of Sport Science, University of the Bundeswehr Munich, Neubiberg, Germany.

^4^ Department of Electrical Engineering, University of the Bundeswehr Munich, Neubiberg, Germany.

**Appendix 1 – SAMHS procedure**

start at MNI coordinate for FDI: x = -34.19, y = -14.33, z = 66.83 (0°)

big 4x4 grid with 10 mm spacing at 0°(points at 45° each)

adjust intensity starting from @ 45% MSO (no EEG; 55% MSO with EEG) to middle point (FDI)

apply 4 single pulses at every grid point

**visible MEPs:**

note grid points with visible MEPs

**no visible MEPs:**

intensity + 10% MSO

repeat 1-4 pulse(s)

per grid point

> 1 valid point:

intensity -5% MSO to 40%

apply 4 pulses at the valid points

chose the point with the most stable and highest amplitudes (3/4) as best

1 valid point:

identify that point as the **hotspot** for big grid

set orientation of big grid hotspot to 0°

small 3x3 grid with 5 mm spacing at the identified spot (45°)

**OPTIONAL**

apply 4 single pulses at every grid point with same intensity

> 1 valid point:

intensity -5% MSO

apply 4 pulses at the valid points chose the point with the most stable and highest amplitudes (3/4) as best

**no visible MEPs:**

intensity + 5% MSO

**visible MEPs:**

note grid points with visible MEPs

1 valid point:

**hotspot** for small grid

repeat 1-4 pulse(s)

per grid point
